# Hereditary Neuropathy with Liability to Pressure Palsy Presenting with Hand Drop in a Young Child

**DOI:** 10.1155/2012/382657

**Published:** 2012-08-16

**Authors:** Inês Sobreira, Cátia Sousa, Ana Raposo, M. Rita Soares, Ana Soudo, Ana Isabel Dias

**Affiliations:** ^1^Department of Paediatrics, Hospital of Divino Espírito Santo, Ponta Delgada EPE, 9500-370 Ponta Delgada, Azores, Portugal; ^2^Physical Medicine and Rehabilitation, Hospital D. Estefânia, Central Lisbon Hospital Center, 1169-045 Lisbon, Portugal; ^3^Department of Neuropediatrics, Hospital D. Estefânia, Central Lisbon Hospital Center, 1169-045 Lisbon, Portugal

## Abstract

Hereditary neuropathy with liability to pressure palsy (HNPP) results from the deletion of the *PMP22* gene in chromosome 17p11.2. Clinically, it presents with painless pressure palsies, typically in the 2nd and 3rd decades of life, being a rare entity in childhood. We present the case study of a six-year-old male child who presented with left hand drop that he kept for over four weeks. Electrophysiological studies suggested HNPP and genetic studies confirmed it. With this paper, we pretend to create awareness to this entity as a diagnosis to be considered in a child with painless monoparesis and to emphasize the importance of electrophysiological studies in the diagnosis.

## 1. Introduction

Hereditary neuropathy with liability to pressure palsy (HNPP) is an autosomal dominant disease characterized by recurrent mononeuropathies with focal sensory or motor disturbance precipitated by minor trauma or compression, similar to those entrapment or compression neuropathies commonly acquired in adulthood, that is, median nerve at carpal tunnel, ulnar at the elbow, and peroneal at fibular head. There is a deletion in chromosome 17p11.2, corresponding to the *PMP22* (peripheral myelin protein 22) codifying gene. This leads to myelin instability with liability to nerve pressure palsy, being the superficial nerves the most affected ones [1–4].

The relation between HNPP and the *PMP22* gene was demonstrated in 1993 [5]; however, other mutations of this gene have been described in Charcot-Marie-Tooth type 1 neuropathy (CMT 1A) [3].

The prevalence of this disease is unknown, mainly due to the inexistence of detailed epidemiological studies, and it has been estimated in 2–5/100000 (European study) and 16/100000 (population study of southwestern Finland) [2, 3].

It usually occurs between the 2nd and 3rd decades of life, being a rare entity in childhood, with about 15 cases published in children less than 10 years old, between 1975 and 2006 [2]. In this age group, the diagnosis is frequently underestimated, mainly when there is no familiar history suggestive of it [1, 2].

The first description of HNPP was made in 1947 by De Jong [6] and it was named “Potato-grubbing disease.” In that publication, he describes a family with recurrent episodes of neuropathy (weakness and numbness of the thumbs, hand and foot drop, in several members of the family and over about four generations) associated with prolonged kneeling during the potato grab. These episodes were initially associated with low vitamin B1 levels. The disease became also known as “Tomaculous Neuropathy” due to the histological alterations on muscular biopsies. 

It is a benign course entity, concomitant with electrophysiological anomalies characterized by mild generalized demyelinating motor and sensory polyneuropathies that may, inclusively, be detected in asymptomatic patients [3, 7]. Most commonly involved nerves include the ulnar, radial, and peroneal ones [1].

Therefore, it is extremely important not only to perform a complete clinical history, but also electrophysiological and genetic studies [4].

In the majority of cases, there is in each episode a complete resolution of the symptoms. However, situations with a precocious onset seem to be associated with a higher risk of recurrence [2].

## 2. Case Report

We present the case study of a 6-year-old Azorean male child, Caucasian, and previously healthy, adopted, with unknown familiar history.

He was admitted in our Emergency Department (Hospital of Divino Espírito Santo of Ponta Delgada-EPE, São Miguel Island, Azores) complaining of left hand drop, which he developed after sleeping in his grandmother's lap.

He denied any past history of trauma, infection, or physical exercise. He had no pain or other symptoms apart from palsy.

Physical examination showed left hand drop, with reduced muscle strength of wrist and fingers extensors. Superficial and deep sensitivities were normal, and deep tendon reflexes were brisk and normal, without muscular atrophies. The remaining physical exam was unremarkable.

Initial therapeutics included vitamin B12 and physiotherapy.

Since there was no improvement after one month, he was sent to a central hospital in Lisbon. At this time, an electrophysiological exam was performed by one of us, confirming not only the radial mononeuropathy but also showing a mild generalized slowing of the sensory conduction velocities with an ulnar conduction block at the elbow, suggesting HNPP.

Genetic study was performed (polymerase chain reaction amplification and multiplex ligation-dependent probe amplification—MLPA) and a deletion in the chromosome 17p11.2 was detected, corresponding to the *PMP22* gene, thus confirming the diagnosis.

The recovery was complete and when seen after 4 months he had no neurological deficits.

## 3. Discussion

With this case study, we pretend to create awareness to HNPP and to the fact that its diagnosis can be missed, mostly during the first decade of life. 

HNPP can present with multiple phenotypes, and it is this clinical heterogeneity that contributes to the difficulty in reaching a diagnosis. The electrophysiological findings observed in HNPP patients (both children and adults) seem to have no relation with clinical severity [3]. 

On the other hand, many relatives clinically do not have symptoms, and only by electrophysiological and genetic studies they were demonstrated to be HNPP affected [3].

In this concrete child, despite the difficulty in obtaining a detailed family history, the electrophysiological study, together with the clinical history, led us to think about HNPP, being this diagnosis posteriorly confirmed by the genetic study.

It has been suggested [2] that the clinical course of this entity in children is characterized by either a complete remission or relapsing paralysis, with a few cases showing a progressive course. 

In fact, early onset HNPP seems to be eventually associated with relapsing and further to the evolution of chronic polyneuropathies [2].

Therefore, it is extremely important to obtain a timely diagnosis, in order to prevent recurrent palsy episodes in the patient and his descendents, namely, in situations that imply prolonged immobilizations with nervous compression (as an example, plaster splints or during prolonged anesthesia).

In spite of being a rare entity in childhood, HNPP hypothesis should be raised upon a child with a focal, acute painless neuropathy. 

As it was demonstrated by this paper, therapeutic approach should include physiotherapeutic treatment, being the recovery slow, but usually total.
